# Multifunctional magnetite nanoparticles to enable delivery of siRNA for the potential treatment of Alzheimer’s

**DOI:** 10.1080/10717544.2020.1775724

**Published:** 2020-06-09

**Authors:** Natalia Lopez-Barbosa, Juan G. Garcia, Javier Cifuentes, Lina M. Castro, Felipe Vargas, Carlos Ostos, Gloria P. Cardona-Gomez, Alher Mauricio Hernandez, Juan C. Cruz

**Affiliations:** a Department of Biomedical Engineering, Universidad de los Andes, Bogota, Colombia; b Institute of Chemistry, CATALAD Research Group, Universidad de Antioquia UdeA, Medellin, Colombia; c School of Medicine, Universidad de Antioquia UdeA, Medellin, Colombia; d Engineering Faculty, Bioinstrumentation and Clinical Engineering Research Group – GIBIC, Bioengineering Department, Universidad de Antioquia UdeA, Medellin, Colombia

**Keywords:** BACE1, siRNA, drug delivery, magnetic nanoparticles, Alzheimer’s disease

## Abstract

Therapeutic drugs for Alzheimer’s disease have been extensively studied due to its recurrence and abundance among neurodegenerative diseases. It is thought that the accumulation of amyloid precursor protein (APP) products, a consequence of an up-regulation of the β-site APP-cleaving enzyme 1 (BACE1), is the main triggering mechanism during the early stages of the disease. This study aims to explore the ability of a multifunctional conjugate based on magnetite nanoparticles for the cellular delivery of siRNA against the expression of the *BACE1* gene. We immobilized the siRNA strand on PEGylated magnetite nanoparticles and investigated the effects on biocompatibility and efficacy of the conjugation. Similarly, we co-immobilized the translocating protein OmpA on PEGylated nanoparticles to enhance cellular uptake and endosomal escape. BACE1 suppression was statistically significant in HFF-1 cells, without any presence of a cytotoxic effect. The delivery of the nanoconjugate was achieved through endocytosis pathways, where endosome formation was likely escaped due to the proton-sponge effect characteristic of PEGylated nanoparticles or mainly by direct translocation in the case of OmpA/PEGylated nanoparticles.

## Introduction

Alzheimer’s disease is the most common cause of dementia and is characterized by causing a decline in cognitive skills that affect the ability of the person to perform everyday activities (Alzheimer’s Association, 2015). Although the exact development of Alzheimer’s disease is still veiled, the accumulation of β-amyloid and tau outside and inside neurons, respectively, are believed to highly contribute to the development of the disease (Vassar et al., 2014). More importantly, vast evidence supports the idea of Alzheimer’s disease develops as a result of an amyloid cascade, in which a deregulated production of β-amyloids is seen as an early step of the pathogenesis (Choi et al., 2014; Herrup, 2015; Karran & De Strooper, 2016; Karch et al., 2018). β-amyloid is a product of two-step proteolysis from the amyloid precursor protein (APP), which is firstly catalyzed by the β-site APP-cleaving enzyme 1 (BACE1) (Corbett et al., 2015). BACE1 is a type I TM aspartic protease with a classical bilobal structure and exhibits two active aspartate motifs (D_93_TG and D_289_SG) (Yan, 2017). Several studies have shown that targeting BACE1 significantly reduces the presence of β-amyloids, serving as a significant option for an Alzheimer’s disease therapy (Wang et al., 2018).

The discovery of interference RNA (iRNA) about 20 years ago (Fire et al., 1998) brought to life the possibility of exploiting gene therapeutics based on gene silencing. Short double-stranded RNA’s (21–23 nucleotides), namely small interference RNA (siRNA), offer a highly specific pathway toward the degradation of target genes by exploiting the natural mechanism of messenger RNA degradation in the cytoplasm (Wittrup & Lieberman, 2015). Nonetheless, effective and targeted cellular delivery and uptake of siRNA remains the main obstacle in the application of siRNA as gene therapy. For instance, naked siRNA exhibits a relatively short half-life due to its rapid degradation by nucleases once it is introduced in the serum (David et al., 2010). Also, cellular uptake is often limited due to its negative phosphate backbone and large molecular weight, making essential the use of a vector that can protect siRNA and promote membrane crossing.

Magnetite nanoparticles offer an interesting solution in which targeted delivery can be easily controlled due to their magnetic responsiveness (Ji et al., 2014), while facilitating imaging and monitoring of the efficacy of the delivery. In addition, magnetite nanoparticles present high chemical stability, biocompatibility, and low toxicity, which makes them attractive for biomedical applications (Muthu & Singh, 2009; Saltan et al., 2011). Due to their high surface energy; however, magnetite nanoparticles tend to rapidly agglomerate under the conditions of biological systems (Ulbrich et al., 2016). For this reason, magnetite nanoparticles are usually conjugated with biological or synthetic polymers that prevent agglomeration and provide suitable routes for siRNA bonding. Polymeric coatings provide a suitable avenue for drug delivery systems due to their slow rate of dissociation that enhances the half-life in the blood compartment of their carry (Otsuka et al., 2012). More specifically, the steric repulsion of poly(ethylene glycol) (PEG)-based conjugates, a consequence of the loss of conformational entropy when close to a foreign moiety, and the low interfacial free energy in aqueous solutions, confer extraordinary physiological properties to the system, such as providing stealth to the nanoparticles and increase their solubility and biocompatibility (Howard et al., 2008; Joralemon et al., 2010; Karakoti et al., 2011). In addition, co-immobilization of cationic peptides and proteins with polymers on magnetite nanoparticles have demonstrated excellent results in terms of cell internalization, endosomal escape abilities, and biocompatibility (Perez et al., 2019; Lopez-Barbosa et al., 2020). Specifically, the OmpA protein, a β - barrel porin that is commonly found in multiple gram-negative bacteria, has shown promising results in the development of drug delivery systems with high cellular uptake and remarkable endosomal escape properties (Lopez-Barbosa et al., 2020).

In this study, we immobilized a specific siRNA modified with thiol groups in the 3′ and 5′ ends on magnetite nanoparticles via coupling with ortho-pyridyl disulfide (OPSS) functionalized polyethylene glycol (PEG) succinimidyl ester (NHS) (OPSS-PEG-NHS). Magnetite nanoparticles were synthesized via thermal decomposition and coprecipitation techniques and characterized in terms of their hydrodynamic diameter, morphology, and surface chemistry. After siRNA immobilization, the cytotoxicity of the conjugates and their ability to reduce the expression of the *BACE1* gene were studied in HFF-1 cells. Additionally, we co-immobilized the translocating protein OmpA on PEGylated nanoparticles to evaluate the impact on cellular uptake, endosomal escape, and biocompatibility in SH-SY5Y cells. This was with the idea of enhancing the penetration of the vehicle and ultimately the amount of delivered cargo. Our study suggests that the obtained multifunctional magnetite nanoparticles serve as a vehicle toward effective BACE1 silencing that can be further exploited as gene therapy for Alzheimer’s disease. Moreover, the co-immobilization of OmpA led to a significant improvement of the endosomal escape abilities of the vehicle without detrimentally impacting biocompatibility. This is critical to assure the feasibility of further *in vivo* studies.

## Methods

### Synthesis of magnetite nanoparticles

Magnetite nanoparticles were obtained by coprecipitation and thermal decomposition techniques. Coprecipitation was performed by dissolving iron (III) chloride hexahydrate (FeCl_3_·6H_2_O) (0.02 mol) and iron (II) chloride tetrahydrate (FeCl_2_·4H_2_O) (0.006 mol) in deionized water (50 mL) after purging it with nitrogen for 20 min. The obtained solution was heated at 90 °C and left still for 10 min. Ammonia solution (5 mL, 25%) was added dropwise to facilitate nanoparticle formation. The ferrofluid was cooled down at room temperature and was washed several times with deionized water and with the help of a strong magnet. Magnetite nanoparticles were dried in an oven to obtain them in powder state.

Thermal decomposition was achieved by decomposing the precursor [Fe(OOC–C_17_H_33_)_3_] (IO-oleate). In brief, sodium oleate (0.017 mol) was dissolved in a solution of ethanol (12 mL), deionized water (9 mL) and hexane (21 mL). FeCl_3_·6H_2_O (0.0059 mol) was added to the solution and heated at 70 °C for 1 h to promote hexane and water evaporation until a waxy oleate was obtained. The waxy byproduct was washed four times with deionized water. Oleic acid (0.0088 mol) was added to the wax and used to dissolve it in 1-octadecene (111 mL). The solution was subjected to a thermal ramp of 3 °C per min until a temperature of 320 °C was reached, the point at which it was left for 3 h. The ferrofluid was cooled down at room temperature and washed several times with ethanol and with the help of a strong magnet.

### Magnetite nanoparticles silanization

Nanoparticle silanization was performed with magnetite nanoparticles obtained by coprecipitation and thermal decomposition techniques. Magnetite nanoparticles from coprecipitation were dispersed in DMSO (10 mL, 0.75 w/v%) to attain a colloid suspension in an aprotic polar solvent. TMAH (10 mL, 1 M) was added to the suspension to facilitate electrostatic and steric stabilization. (3-aminopropyl) triethoxysilane (APTES) (5 mL) was added to the solution and mechanically stirred for 24 h. Modified nanoparticles were washed several times with hexane and ethanol and with the help of a strong magnet.

Magnetite nanoparticles from thermal decomposition (35 mL) were dispersed in hexane (80 mL). Glacial acetic acid (50 μL) was added to the solution to promote the cross-linking reaction. APTES (6 mL) was added to the solution and mechanically stirred for 24 h. Modified nanoparticles were washed several times with hexane and ethanol and with help of a strong magnet.

### Silanized nanoparticles characterization

X-ray diffraction (XRD) was carried out in a PANalytical Empyrean diffractometer using CuKαradiation (λ = 1.5406 Å) and was used to characterize the synthesis and silanization of magnetite nanoparticles via coprecipitation and thermal decomposition. Raman spectroscopy of coprecipitated magnetite nanoparticles was recorded to observe whether the maghemite phase was formed in a high-resolution Labram HR spectrometer (Horiba, Piscataway, NJ). Scanning electron microscopy (SEM) (JSM-6490LV JEOL) and transmission electron microscopy (TEM) (FEI Tecnai G2 F20 Super Twin TMP) were used to observe nanoparticle size and morphology after silanization. TEM images were recorded after the suspension of the nanoparticles in ethanol. Electron diffraction patterns and inverse Fourier transform were applied with the software Gatan Digital Micrograph® to analyze the crystalline planes of the nanoparticles. The hydrodynamic diameter of silanized nanoparticles was calculated by Dynamic Light Scattering (DLS) technique (Nano ZS Zetasizer, Malvern Instruments, UK) and Small-angle X-ray Scattering (SAXS). Fourier Transform Infrared (FT-IR) spectra were recorded prior and after the silanization process between 3600 and 450 cm^−1^ and with a resolution of 4 cm^−1^.

The number of amine groups in silanized nanoparticles was confirmed by Thermogravimetric analysis (TGA) (TA Instruments, Lindon, UT). TGA was performed by ramping the temperature at a rate of 10 °C/min from 25 to 800 °C.

### siRNA immobilization

Small interference RNA (siRNA) modified with thiol groups in the 3′ and 5′ ends (Sigma-Aldrich, St. Louis, MO) was immobilized on magnetite nanoparticles via coupling with ortho-pyridyl disulfide (OPSS) functionalized polyethylene glycol (PEG) succinimidyl ester (NHS) (OPSS-PEG-NHS). Briefly, OPSS-PEG-NHS (150 mg) was dissolved in dimethylformamide (DMF) (2 mL), which was previously purged with nitrogen. Silanized nanoparticles from either coprecipitation or thermal decomposition technique (10 mg) were added to the solution with further mechanical agitation for 24 h. Modified nanoparticles (Fe_3_O_4_-PEG-OPSS) were washed several times with ethanol and acetone to remove the excess of OPSS-PEG-NHS. Fe_3_O_4_-PEG-OPSS were washed with milli Q water and placed in RNase-free buffer solution (1 mL) with further sonication for 30 min. Treated Fe_3_O_4_-PEG-OPSS were placed in a dialysis membrane (10,000 COMW) and submerged in a Ringer’s lactate solution.

In parallel, dithiothreitol (DTT) (0.04 mmol) was dissolved in RNase-free buffer solution (0.5 mL) purged with nitrogen. siRNA (50 mmol) was added to RNase-free buffer solution (5 μL) purged with Nitrogen. DTT solution (2 μL) was added to siRNA solution and stirred for 30 min under an inert atmosphere. siRNA solution was placed in the dialysis membrane with Fe_3_O_4_-PEG-OPSS. Ringer’s lactate solution was changed every two hours to ensure maximum DTT removal. The reaction was carried out for a total of 6 h. When the reaction was completed, immobilized nanoparticles (Fe_3_O_4_-PEG-siRNA) were centrifugated at 2500 rpm for 5 min and washed several times with DNase- and RNase-free water. Fe_3_O_4_-PEG-siRNA were resuspended in carboxymethyl cellulose (CMC) (1 mL, 1% (v/v)).

### Cell lines, culture conditions, and subcultures

The HFF-1 human fibroblast cell line (HFF-1-ATCC^®^ SCRC-1041™) and the SH-SY5Y human neuroblastoma (ATCC^®^ SH-SY5Y) were obtained from the ATCC. Both cell lines were cultured in DMEM supplemented with 15% heat-inactivated FBS and 1% penicillin and streptomycin at 37 °C in humidified air with 5% CO_2_. HFF-1 and SH-SY5Y cells were periodically passed and tested for contaminations.

### MTT assay and quantification

Cytotoxicity of the nanoparticles was tested via MTT assay. Briefly, HFF-1 cells were plated in 96 well culture plates (200,000 cells/100 μL/well) and incubated at 37 °C, 5% CO_2_ for 24 h. Culture media was removed from wells and DMEM 1% penicillin/streptomycin (90 μL) (without FBS) was added to each well. Fe_3_O_4_-PEG-siRNA (100 μL, 100 μg/mL) was added by triplicate and incubated at 37 °C, 5% CO_2_ for 1, 2, and 7 days. Further, MTT (10 μL, 5 mg/mL) was added to each well and incubated for an additional 2 h. Culture media was replaced with DMSO to dissolve formazan crystals. Absorbance was read at 595 nm in a Multiwell plate reader and compared with positive controls (cells cultured in DMSO).

Data normalization was established with the Shapiro–Wilk test. Data sets are shown as an average with their standard error. Viability tests results of treated cells were compared with the negative control and analyzed using a one-way ANOVA with a 95% significance level (*α* = 0.05).

### BACE1 expression in HFF-1 cells

300,000 HFF-1 cells/mL were seeded in 6 well plates in DMEM supplemented medium and incubated at 37 °C, 5% CO_2_ for 24 h. Cells were exposed to Fe_3_O_4_ nanoparticles, Fe_3_O_4_-PEG-OPSS and Fe_3_O_4_-PEG-siRNA, respectively. Cells exposed to DMEM supplemented with CMC 1% (v/v) were used as positive controls, while cells without any exposure were used as negative controls. CMC was used before cell exposure to assure that nanoparticles were well suspended in the medium.

Quantitative real-time PT-PCR was used to quantify the relative expression of BACE1 in HFF-1 cells. Briefly, RNA was extracted from control and treated HFF-1 cells using TRIZOL reagent and following the standard phenol-chloroform extraction method. Quality of RNA extraction was confirmed via agarose gel electrophoresis and spectrophotometry with a 260 nm: 280 nm ratio using a NanoDrop Spectrophotometer (Thermo Fisher Scientific, Waltham, MA). Reverse transcription reactions were performed under MMLV-RT and random hexamers, following the manufacturer’s protocol. qRT-PCR was carried out in triplicates and using SyBR green (Applied Biosystems, Foster City, CA). Primers for BACE1 were as follows: F: 5′-ACCAACCTTCGTTTGCCCAA-3′ and R: 5′-TCTCCTAGCCAGAAACCATCAG-3′. β-actin was used as the housekeeping gene for normalization purposes. Polymerase activation was achieved by 10 min heating at 95 °C followed by 40 cycles of denaturation and 1 min of combined stringed annealing/extension at 60 °C.

Statistical analysis was based on the Mann-Whitney test for unpaired analyses by comparing the average expression between classes. Results were considered statistically significant if their *p*-value was below .05.

### OmpA overexpression in Escherichia coli

Overexpression of OmpA protein in *E. Coli* was accomplished in accordance with the protocol by Gonzalez Barrios et al. (Aguilera Segura et al., 2014). *Escherichia coli* K-12 W3110/pCA24N OmpA + was cultured in LB agar plates containing yeast extract (5 g/L), bacto tryptone (10 g/L), NaCl (10 g/L) and chloramphenicol (50 μg/mL), overnight and at 37 °C. Further, inoculation of a colony was attained in LB medium with chloramphenicol (50 mL) and was incubated for 16 h at 37 °C, 250 rpm. In addition, a fresh LB medium (19.5 mL) was inoculated with the previous culture (500 μL) for 16 h at 37 °C, 250 rpm. Cultures were grown until reaching an optical density at 600 nm (OD_600nm_) of 0.7. At this point, isopropylthio-β-galactoside (IPTG, 95%, Sigma-Aldrich) (2 mM) was added to induce OmpA expression. IPTG exposure was performed for 3 h.

### OmpA purification and characterization

A pellet of OmpA overexpressed *E. Coli* was obtained from the culture and prepared for purification. A lysis buffer was added to the pellet in a ratio of 4 mL per gram of pellet and sonicated for 40 min at 37% amplitude in ice. The obtained solution was centrifuged at 13,000 rpm and 4 °C for 15 min and the supernatant was recovered. Purification was attained by exposing the supernatant to the Dynabeads TALON kit (Invitrogen, Waltham, MA) since OmpA protein was cloned with an additional histidine tail. The presence of purified OmpA was verified with sodium dodecyl sulfate polyacrylamide gel electrophoresis (SDS-PAGE), which showed a single 31 kDa band that agrees well with the molecular weight of OmpA. Concentration was measured with a NanoDrop Spectrophotometer (Thermo Fisher Scientific) at 280 nm.

### Synthesis of OmpA/PEGylated nanoparticles

An amine-terminated version of the PEG molecule (creative PEG, Chapel Hill, NC) was implemented to facilitate the synthesis of rhodamine B-labeled PEGylated nanoparticles and OmpA/PEGylated nanoparticles. The amine-terminated version of the PEG molecule exhibits the same chemistry and molecular weight of the thiolated version (i.e. OPSS-PEG). This was to assure that the two sets of experiments were comparable.

Amino terminated PEG (300 mg) was dissolved in dimethylformamide (DMF) (4 mL), which was previously purged with Nitrogen to desorb Oxygen. Silanized nanoparticles obtained by coprecipitation (20 mg) were added to the solution with further mechanical agitation for 24 h to complete the conjugation. The obtained PEGylated nanoparticles (Fe_3_O_4_-PEG-NH_2_) were washed several times with ethanol and type I water (Ultrapure water with a resistivity > 18 MΩ cm, and conductivity < 0.056 µS/cm) to remove the excess of amine-terminated PEG. PEGylated nanoparticles were then resuspended in 20 mL of type I water and stored at 4 °C. Similarly, for the OmpA/PEGylated nanoparticles synthesis, PEGylated nanoparticles (10 mL, 1 mg/mL) were sonicated for 5 min at 40 kHz frequency and 38% amplitude. Subsequently, 1 mL of glutaraldehyde in type I water solution (2% (v/v)) was added and left at constant mechanical stirring (200 rpm) for 1 h. Then, OmpA solution (10 mL, 2 mg/mL) was added to the previous glutaraldehyde activated PEGylated nanoparticles and left reacting under constant mechanical agitation for 24 h. OmpA/PEGylated nanoparticles were washed several times with type I water, resuspended, and stored at 4 °C.

### Labeling of PEGylated nanoparticles and OmpA/PEGylated nanoparticles with rhodamine B

PEGylated nanoparticles and OmpA/PEGylated nanoparticles were labeled with rhodamine B for cell penetration and endosomal escape analysis under confocal microscope observation. This was achieved by forming amide bonds between the carboxyl groups of rhodamine B and the free amine groups of conjugated OmpA and PEG, respectively. Briefly, 30 mg of rhodamine B (95%, Sigma-Aldrich, USA), 12.3 mg of N-[3-dimethylammino)-propyl]-N′-ethyl carbodiimide hydrochloride (EDC, 98%, Sigma-Aldrich, USA) and 7.4 mg of N-hydroxy succinimide (NHS, 98%, Sigma-Aldrich, USA) were dissolved in 2 mL of NN-dimethylformamide (DMF, 99.8%, Sigma-Aldrich, USA) and diluted in 3 mL of type I water. The resulting solution was heated to 37 °C under continuous magnetic stirring (200 rpm) for 15 min to activate the carboxyl groups for conjugation. Then, rhodamine B solution was added to 40 mL of an aqueous suspension of PEGylated nanoparticles and OmpA/PEGylated nanoparticles in type I water (2.5 mg/mL) and sonicated for 5 min at 40 kHz frequency and 38% amplitude. The reaction mixture was left under constant mechanical stirring (200 rpm) at room temperature and complete darkness (to avoid photobleaching) for 24 h. The obtained rhodamine B-labeled nanoparticles were washed several times with type I water. Finally, rhodamine B-labeled nanoparticles were sonicated for 10 min (frequency 40 kHz, amplitude 38%) and stored at 4 °C under complete darkness until further use.

### Hemolysis assay

Hemolysis of magnetite nanoparticles, PEGylated nanoparticles, and OmpA/PEGylated nanoparticles was tested on erythrocytes isolated from freshly drawn blood of a healthy human donor. The blood was collected in a vacutainer tube with EDTA to avoid aggregation. The sample was centrifuged at 1800 rpm for 5 min at room temperature. Subsequently, the supernatant with the plasma was discarded and then, the precipitate containing the erythrocytes was resuspended and washed five times with NaCl solution (0.9% (w/v)). Erythrocytes stock was prepared by adding 1 mL of isolated erythrocytes (4.3 × 10^6^ erythrocytes/μL) in 9 mL of PBS (1X). Each sample was tested in serial dilutions from 100 to 12.5 μg/mL prepared by mixing concentrated stocks with PBS (1X). Triton X-100 (1% (v/v)) and PBS (1X) were used as positive and negative controls, respectively. 100 μL of erythrocytes was placed in a 96-well microplate and exposed to 100 μL of the different treatments previously sonicated for 5 min at 40 kHz frequency and 38% amplitude. Next, samples were gently resuspended and incubated at 37 °C, 5% CO_2_ for 1 h. After the exposure time, samples were centrifuged at 1800 rpm for 5 min and 100 μL of each supernatant was placed in a 96-well microplate and read at 450 nm in a microplate reader. Finally, the hemolysis percentage was calculated by subtracting the absorbance of the negative control from the test sample and dividing by the difference of the controls (positive control − negative control).

### Platelet aggregation assay

Platelet aggregation tendency of magnetite nanoparticles, PEGylated nanoparticles, and OmpA/PEGylated nanoparticles were tested on platelets isolated from freshly drawn blood of a healthy human donor. The blood sample was collected in a vacutainer tube with sodium citrate as an anticoagulant to avoid platelet aggregation. The sample was centrifuged at 1000 rpm for 15 min at room temperature to obtain the platelet-rich plasma (PRP). Each sample was tested in serial dilutions from 100 to 12.5 μg/mL prepared by mixing concentrated stocks with PBS (1X). Thrombin (6 U) was used as a positive control, while PBS (1X) served as the negative one. 50 μL of PRP were seeded in a 96-well microplate and exposed to 50 μL of the different treatments previously sonicated for 5 min at 40 kHz frequency and 38% amplitude. Samples were incubated at 37 °C, 5% CO_2_ for 5 min. Finally, 50 μL of each supernatant was transferred to a 96-well microplate and read at 620 nm in a microplate reader.

### LDH cytotoxicity assay

Cytotoxicity of magnetite nanoparticles and OmpA/PEGylated nanoparticles was tested via LDH assay. Briefly, SH-SY5Y cells were plated in 96-well culture plates (5000 cells/100 mL/well) and incubated at 37 °C, 5% CO_2_ for 24 h. In parallel, serial dilutions (i.e. 100–12.5 μg/mL) were prepared by mixing the concentrated stocks with DMEM 1% penicillin/streptomycin (without FBS). Triton X-100 (1% (v/v)) was used as positive control and DMEM media as the negative one. After the 24 h, supplemented culture media was removed from wells and replaced with 100 μL of the different treatments previously sonicated for 5 min at 40 kHz frequency and 38% amplitude. Samples were incubated at 37 °C, 5% CO_2_ for 1 and 2 days. Further, 50 μL of each supernatant were extracted and placed in 96-well microplates with 50 μL of the reaction mixture (Cytotoxicity Detection Kit (LDH), Roche, Switzerland) and left to react under mechanical stirring (50 rpm) at room temperature and complete darkness for 15 min. Finally, absorbance was read at 490 nm in a microplate reader.

### Endosomal escape of PEGylated nanoparticles and OmpA/PEGylated nanoparticles in SH-SY5Y cells

Endosomal escape of PEGylated nanoparticles and OmpA/PEGylated nanoparticles was assessed by colocalization of Lysotracker Green DND-26 (Thermo Fisher, USA) and rhodamine B-labeled nanoparticles after internalization into SH-SY5Y cells. Briefly, 50,000 SH-SY5Y cells were seeded in a sterile glass slide previously placed in a 24-well microplate and incubated at 37 °C, 5% CO_2_ for 24 h. Supplemented culture media was removed and replaced with 500 μL of rhodamine B-labeled nanoparticles solution (50 μg/mL) previously sonicated for 5 min at 40 kHz frequency and 38% amplitude. Cells exposed to labeled-nanoparticles were incubated at 37 °C, 5% CO_2_ for 2 h. After incubation, cells were washed 3 times with PBS (1X) and then, exposed to 500 μL of DMEM solution containing Hoechst 33342 (Thermo Fisher) (1:1000) and Lysotracker Green DND-26 (Thermo Fisher) (1: 10,000) for 10 min. The images were obtained using an Olympus FV1000 confocal laser scanning microscope (CLSM) with a PlanApo 60x, 1.35 NA oil-immersion objective at excitation/emission wavelengths of 358 nm/461 nm, 488 nm/520 nm, and 546 nm/575 nm for detection of nuclei, endosomes, and nanoparticles, respectively. Colocalization over space was analyzed using the plugin Coloc 2 in the Fiji software^®^ by simultaneously looking at the green and red channels.

## Results

### Nanoparticles characterization

**Figure 1.**
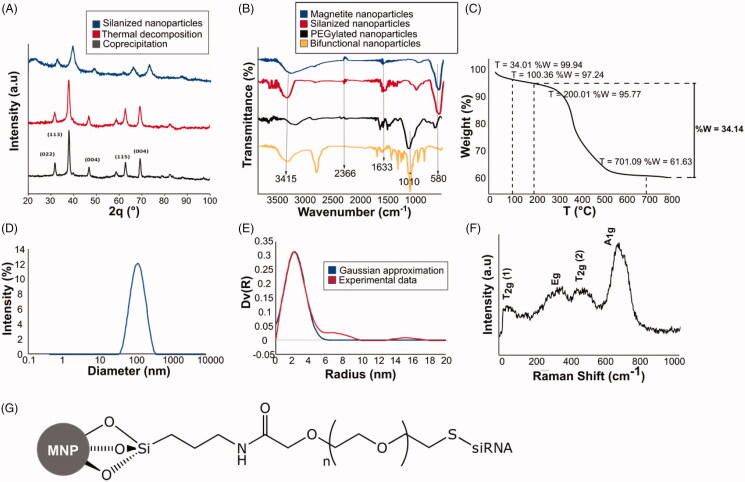
Characterization of magnetite and silanized nanoparticles. (A) X-ray diffraction (XRD) patterns of magnetite nanoparticles obtained by (red) coprecipitation and (black) thermal decomposition techniques, and (blue) after silanization. The nanoparticles exhibit and inversed spinel crystalline structured, which is translated into a fd3m spinel-type after silanization with APTES. (B) Fourier Transform infrared (FT-IR) analysis of the reaction steps for the functionalization of the magnetite nanoparticles. (blue) Magnetite nanoparticles, (red) silanized nanoparticles, (black) PEGylated nanoparticles and (yellow) bifunctional nanoparticles. Bands at 3415 cm^−1^ and 960 cm^−1^ correspond to the stretching and bending of the O–H bond at the surface of the nanoparticles. Bands at 1633 cm^−1^ and 580 cm^−1^ correlate with the bending of the H–O–H and the vibration of the Fe–O bond, respectively. The stretching of the Si–O–Si bond is identified in the 1010 cm^−1^ band that appears after silanization. The stretching of the amide C = O bond is observed with the appearance of a new peak around 1741 cm^−1^ after PEGylation. CH stretches of the heteroaromatic ortho-pyridyl ring appear at 2800 cm^−1^ in the bifunctional nanoparticles. (C) Thermogravimetric analysis (TGA) of silanized nanoparticles. Three weight loss steps are identified in the graph and are attributed as follows: weight loss step at 100 °C to water loss, weight loss step at 200 °C to physically absorbed APTES, and weight loss step at 701 °C to silanized APTES species. (D, E) Hydrodynamic diameter of silanized nanoparticles obtained by (D) dynamic light scattering (DLS) and (E) small-angle X-ray scattering (SAXS) techniques. In (E), the red curve corresponds to experimental data, while the blue curve to a theoretical Gaussian approximation. (F) Raman spectroscopy of the magnetic nanoparticles. Magnetite composition is identified due to the appearance of the four phonon bands at room temperature. T_2g_, A_1g_ and E_g_ modes are identified in accordance with the quasi-molecular description of the spinel tetrahedron. (G) Chemical structure of the PEGylated conjugate after immobilization of siRNA.

XRD measurements were performed prior to and after the silanization of the magnetite nanoparticles. Identified peaks in Figure 1(A) allowed to confirm the effective synthesis of magnetite nanoparticles by both coprecipitation and thermal decomposition techniques, showing a crystalline structure of inverse spinel, which is characteristic of magnetite nanoparticles (Fe_3_O_4_) (JCPDS 15-8743). Lanthanum hexaboride (LaB_6_) was used as a correction factor to obtain the approximate size of the ordered crystalline domains by using Scherrer equation (Equation (1); Hui et al., 2015; Tatarchuk et al., 2017) as follows:

(1) $$\tau =\frac{K\lambda }{\beta \;\text{cos}\;\theta }$$

Where K corresponds to the shape factor, λ to the X-ray wavelength, β to the line broadening at half the maximum intensity (FWHM), and θ to the Bragg angle. The size of the obtained magnetite nanoparticles ranged between 6 and 10 nm. XRD patterns vary slightly after silanization with APTES (Figure 1(A)). As observed, the most evident changes occurred for 2θ angles in the range between 20 and 25°, arising a spinel-type crystal structure (Fd3m).

To be certain about the composition of the magnetic nanoparticles, Raman Spectroscopy was recorded and is shown in Figure 1(F). Raman spectroscopy allows the differentiation between magnetite and maghemite compounds due to the four characteristic phonon bands encountered in magnetite at room temperature. As observed in the spectrum, the phonon bands at 192 and 492 cm^−1^ are of the T_2g_ symmetry and arouse from the asymmetric stretch of Fe and O bonds and the translational motion of the whole system. The phonon band at 681 cm^−1^ is of A_1g_ nature and is originated from the symmetric stretch of the oxygen atoms in the Fe–O bond; and the phonon band at 372 cm^−1^ is an *E_g_* specie, a consequence of the symmetric bends of oxygen with respect to Fe (Shebanova & Lazor, 2003).

DLS and SAXS techniques were used to determine the hydrodynamic diameter of silanized nanoparticles (Figure 1(D,E)). Results obtained from both techniques are significantly different, being DLS results one order of magnitude greater. For instance, DLS measurements were performed with silanized nanoparticles suspended in ethanol, while SAXS experiments were performed with dry samples. Although the high conductivity of ethanol is sufficient to suppress the formation of a significant electrical double layer (Vello et al., 2017), agglomeration of the nanoparticles prior and during the measurement is inevitable.

The functionalization reaction steps prior to the addition of siRNA were monitored through FT-IR analysis (Figure 1(B)) at the stages of bare magnetite nanoparticles, silanization with APTES, PEGylation, and bifunctionalization of the nanoparticles. The presence of the magnetite nanoparticles was confirmed through the bands at 3415 cm^−1^ and 960 cm^−1^, which correspond to the stretching and bending of the O–H bond at the surface of the nanoparticles and the bands at 1633 cm^−1^ and 580 cm^−1^, which correlate with the bending of the H–O–H and the vibration of the Fe–O bond, respectively. The stretching of the Si–O–Si bond is identified in the 1010 cm^−1^ band that appears after silanization. The stretching of the amide C = O bond is observed with the appearance of a new peak around 1741 cm^−1^ after PEGylation. CH stretches of the heteroaromatic ortho-pyridyl ring appear at 2800 cm^−1^ in the bifunctional nanoparticles.

TGA was used to evaluate the extend of silanization and amine groups in silanized nanoparticles (Figure 1(C)). Three main weight loss steps can be identified at 100, 200, and 701 °C. The first weight loss step is attributed to water loss, the second weight loss step is highly likely to be associated with physically absorbed APTES species while the third weight loss step corresponds to the decomposition of APTES molecules bound to the surface of the nanoparticles (Qiao et al., 2015).

**Figure 2.**
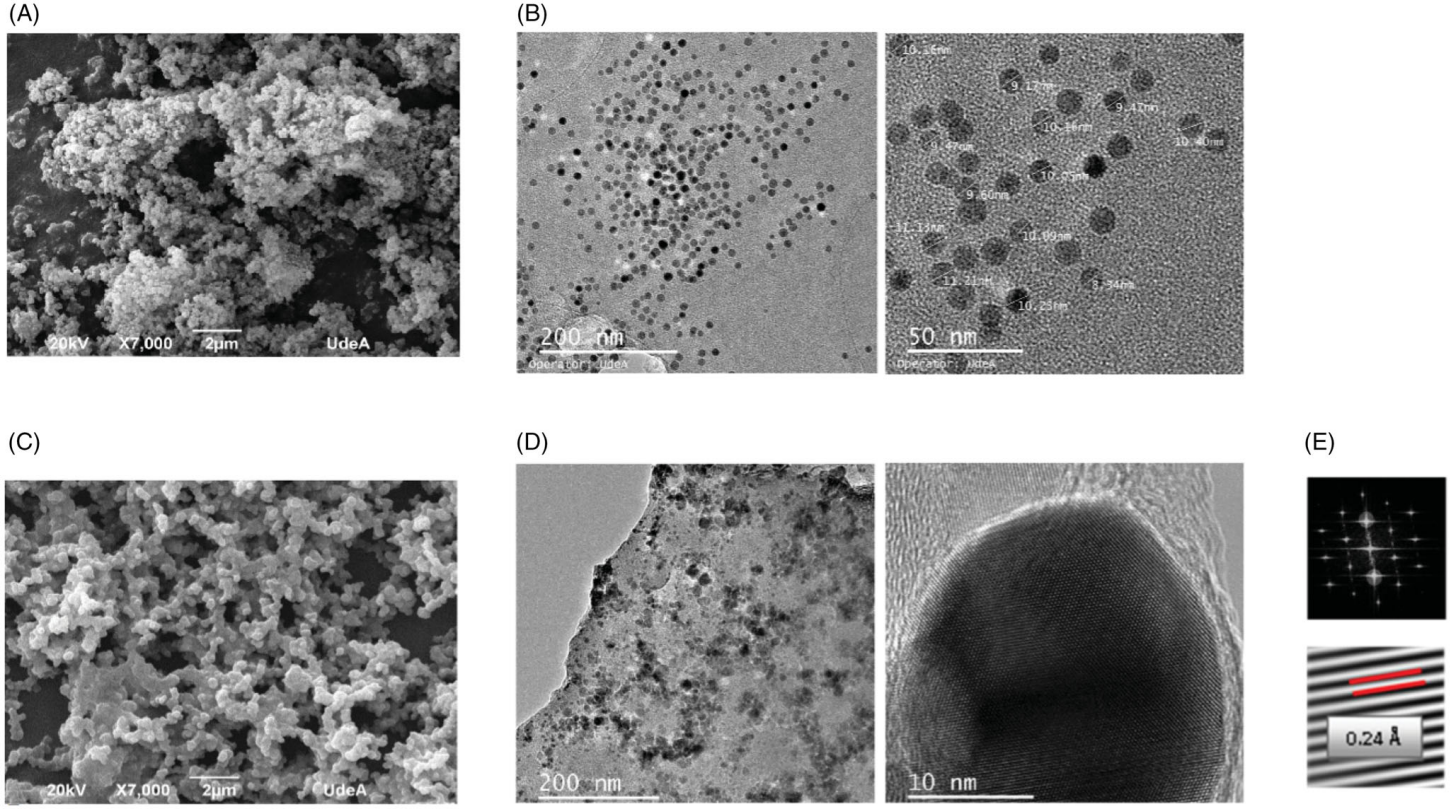
Characterization of the morphology of the nanoparticles prior and after silanization. Scanning electron microscopy (SEM) micrographs of silanized magnetite nanoparticles obtained from (A) thermal decomposition and (C) coprecipitation. Transmission electron microscopy (TEM) images of magnetite nanoparticles (B) prior and (D) after silanization. (E) Electron diffraction patterns after silanization allowed to observe the inverse spinel structure characteristic of magnetite unit cell, featuring lattice fringes with a d-spacing of 0.24 Å, consistent with 111 planes.

SEM micrographs were recorded to identify the morphology of the nanoparticles synthesized by both coprecipitation and thermal decomposition prior to and after APTES silanization (Figures 2(A,C)). This process is highly likely to induce agglomeration of the nanoparticles mainly due to the polycondensation of silicon precursors (Figure 2(D)). Interestingly, nanoparticles obtained from the coprecipitation method seemed to maintain a more uniform spherical morphology (Figure 2(C)) than those from thermal decomposition (Figure 2(A)).

TEM images were recorded prior to and after silanization to observe morphological changes of magnetite nanoparticles due to the process. In addition, electron diffraction (ED) patterns of silanized nanoparticles allowed us to observe the stability of magnetite crystals after silanization. Figure 2(B) shows that the obtained nanoparticles are highly homogeneous in size and morphology prior to the silanization process, and their size agrees well with that obtained with the Scherrer equation (Equation (1)). ED patterns of silanized nanoparticles (Figure 2(E)) feature lattice fringes with a d-spacing of 0.24 Å, which is consistent with the (111) planes.

### BACE1 expression in HFF-1 cells

**Figure 3.**
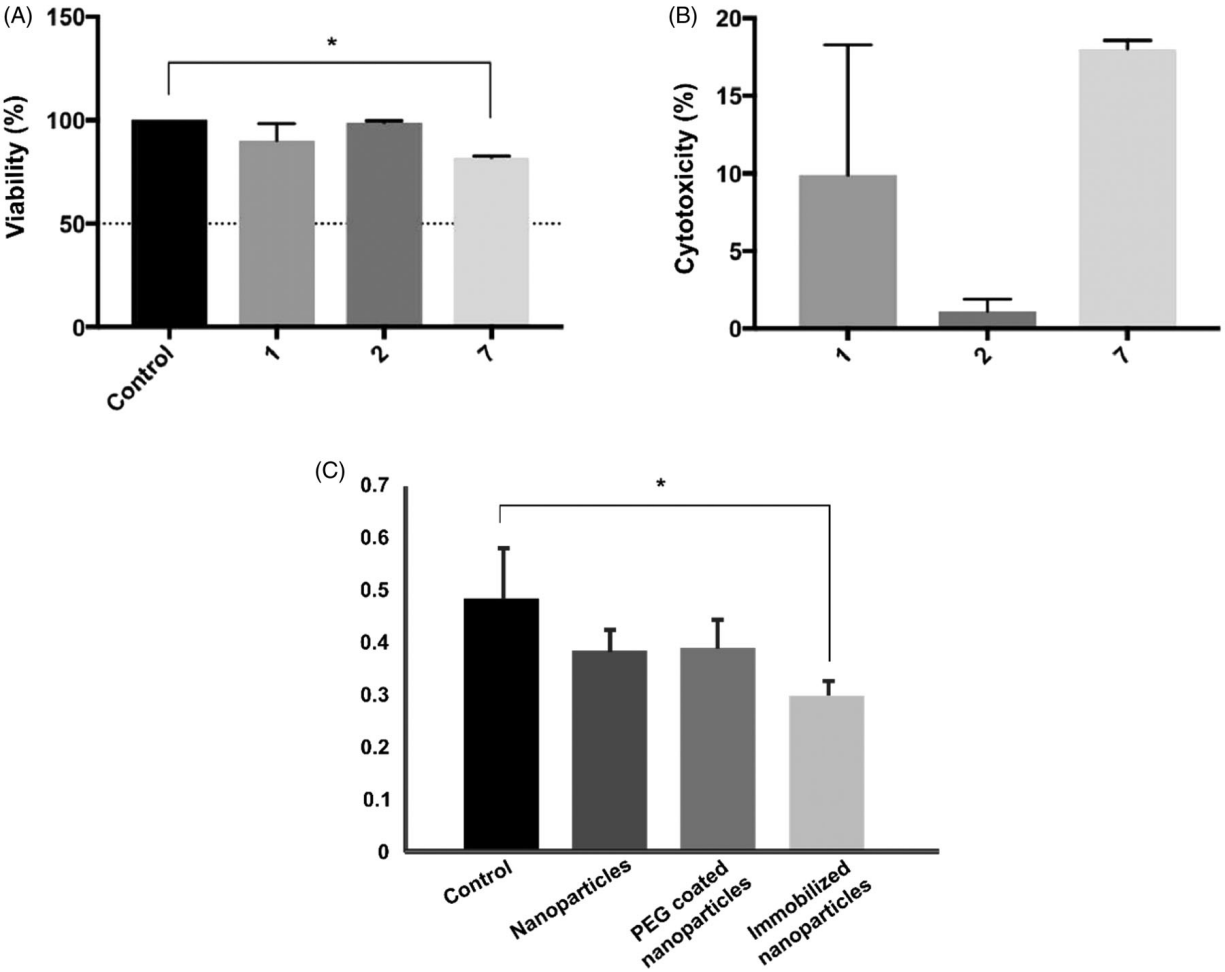
(A) Viability and (B) cytotoxicity effects of immobilized siRNA nanoparticles after 1, 2, and 7 days. One-way ANOVA with a *p*-value < .05 (*) +was used to determine statistically significant difference and was only found between the positive control and results after 7 days of exposure. Nonetheless, viability levels were always kept above 80%, indicating acute and chronic biocompatibility. (C) BACE-1 relative expression in HFF-1 cells after cellular treatment with bare magnetite nanoparticles, PEG coated nanoparticles and immobilized siRNA nanoconjugates. Kruskal–Wallis analysis was used to determine significant difference (*) *p* < .05.

Small interference RNA (siRNA) modified with thiol groups in the 3′ and 5′ ends was immobilized on magnetite nanoparticles obtained through thermal decomposition via coupling with ortho-pyridyl disulfide (OPSS) functionalized polyethylene glycol (PEG) succinimidyl ester (NHS) (OPSS-PEG-NHS). Prior to the evaluation of the system as a suppressor of BACE1 expression in HFF-1 cells, cytotoxicity studies via standard viability MTT assay were conducted. HFF-1 cells cultured in DMSO and DMEM medium were used as positive and negative controls, respectively. Viability was monitored after 1, 2, and 7 days as shown in Figure 3(A). Recorded results show that viability levels were maintained above 80% even after 7 days of exposure, with values approaching 90.09 ± 4.84%, 98.88 ± 0.56%, and 81.99 ± 0.39% for days 1, 2 and 7, respectively. In addition, statistical analysis showed that there is only a significant difference between the viability of the positive control and cells exposed to immobilized siRNA nanoconjugates after 7 days of exposure. Cytotoxicity was calculated from viability results and is shown in Figure 3(B). Cytotoxicity percentages were found to be 9.91 ± 4.84%, 1.12 ± 0.56%, and 18.01 ± 0.39% after 1, 2, and 7 days, respectively. This indicates that immobilized siRNA nanoparticles at a concentration of 100 μg/mL exhibit both acute and chronic biocompatibility in HFF-1 cells.

Quantitative RT-PCR was used to determine the effectiveness of immobilized siRNA in silencing *BACE1* gene expression after incubation for 24 h. Bare magnetite nanoparticles and PEG-coated nanoparticles were also measured to guarantee that silencing was only due to the presence of the siRNA strand. Figure 3(C) shows the relative BACE1 expression found in each of the treatments. Only immobilized siRNA nanoconjugates showed a statistically significant difference with HFF-1 cells in DMEM medium, thereby suggesting that BACE1 expression was successfully decreased due to the silencing by the siRNA.

### Internalization and endosomal escape

**Figure 4.**
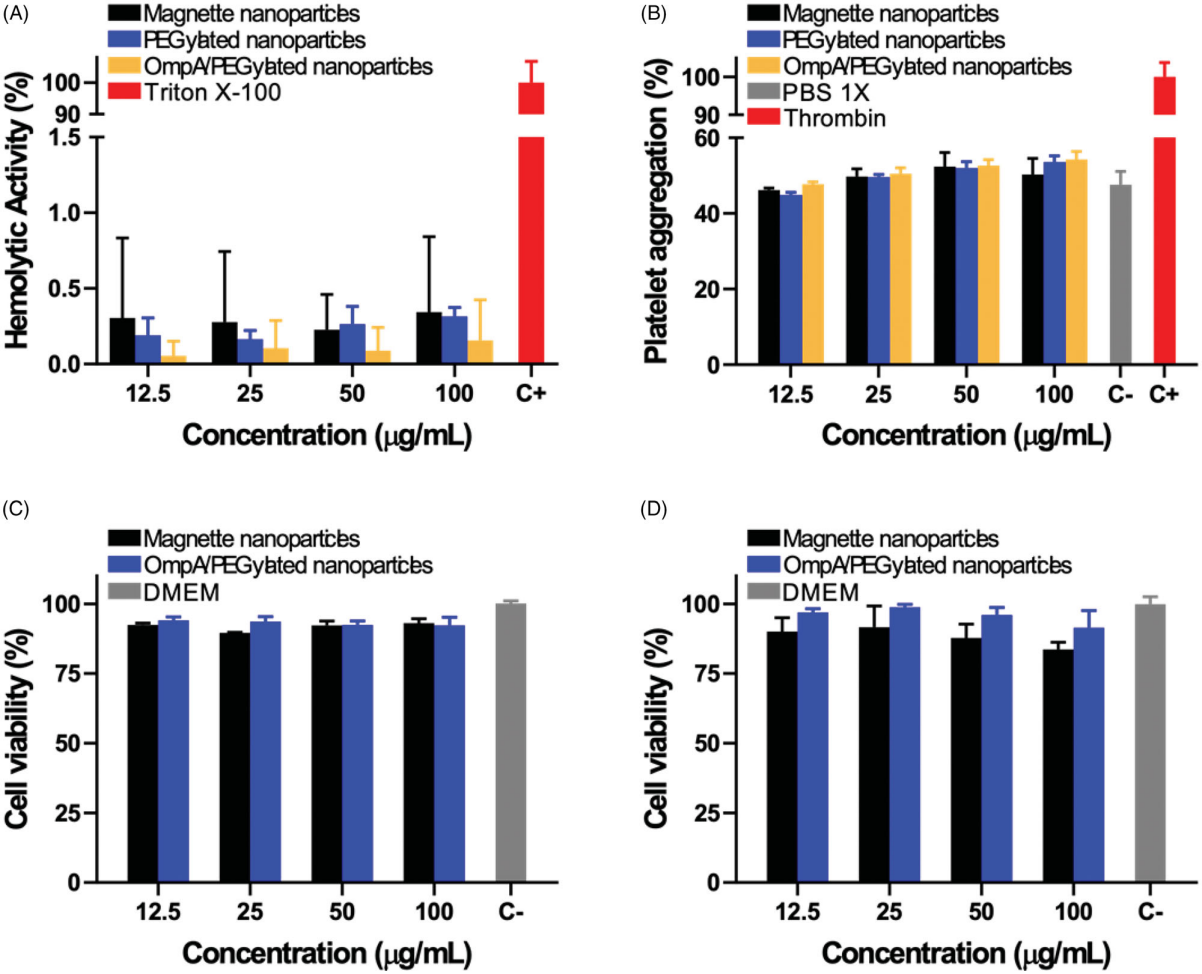
(A) Assessment of the hemolytic effect of magnetite nanoparticles, PEGylated nanoparticles, and OmpA/PEGylated nanoparticles. Hemolysis assay shows an average hemolytic effect below 1% in all cases. (B) Platelet aggregation tendency of magnetite nanoparticles, PEGylated nanoparticles, and OmpA/PEGylated nanoparticles. Results show that there is no significant increase in the platelet aggregation of the different treatments compared to the negative control, PBS (1X). Cytotoxicity effects of OmpA/PEGylated nanoparticles after 1 (C) and 2 days (D). For all the tested concentrations, the observed viability levels were always above 90%, thereby indicating that the co-immobilization of OmpA has no significant impact on the high biocompatibility of the PEGylated nanoparticles.

Further studies were carried out to verify biocompatibility, internalization, and endosomal escape abilities of PEGylated nanoparticles and OmpA/PEGylated nanoparticles. For this, the nanovehicles were delivered in SH-SY5Y cells. In addition, since the main administration strategy of the developed vehicles is most likely to be intravenous (IV), we analyzed their impact on red blood cells as well as the potential thrombogenic activity. Figure 4(A,B) shows the results obtained from hemolysis and platelet aggregation assays, respectively. All the treatments showed a hemolysis percentage below 1%, thereby corroborating high hemocompatibility. Moreover, even at the highest evaluated concentrations, platelet aggregation percentage remained below about 50% and showed no statistically significant difference with respect to the aggregation level of the negative control, PBS (1X). These results are consistent with previous reports that showed improved hemocompatibility and a significant decrease in thrombogenicity for different PEGylated nanomaterials (Ilinskaya & Dobrovolskaia, 2016). We also evaluated cytotoxicity via LDH assay. Cell viability was studied after 1 and 2 days of exposure as shown in Figure 4(C,D), respectively. The obtained results show that viability levels of both PEGylated and OmpA/PEGylated nanoparticles in all the evaluated concentrations remained above 90% even after 2 days of exposure.

**Figure 5.**
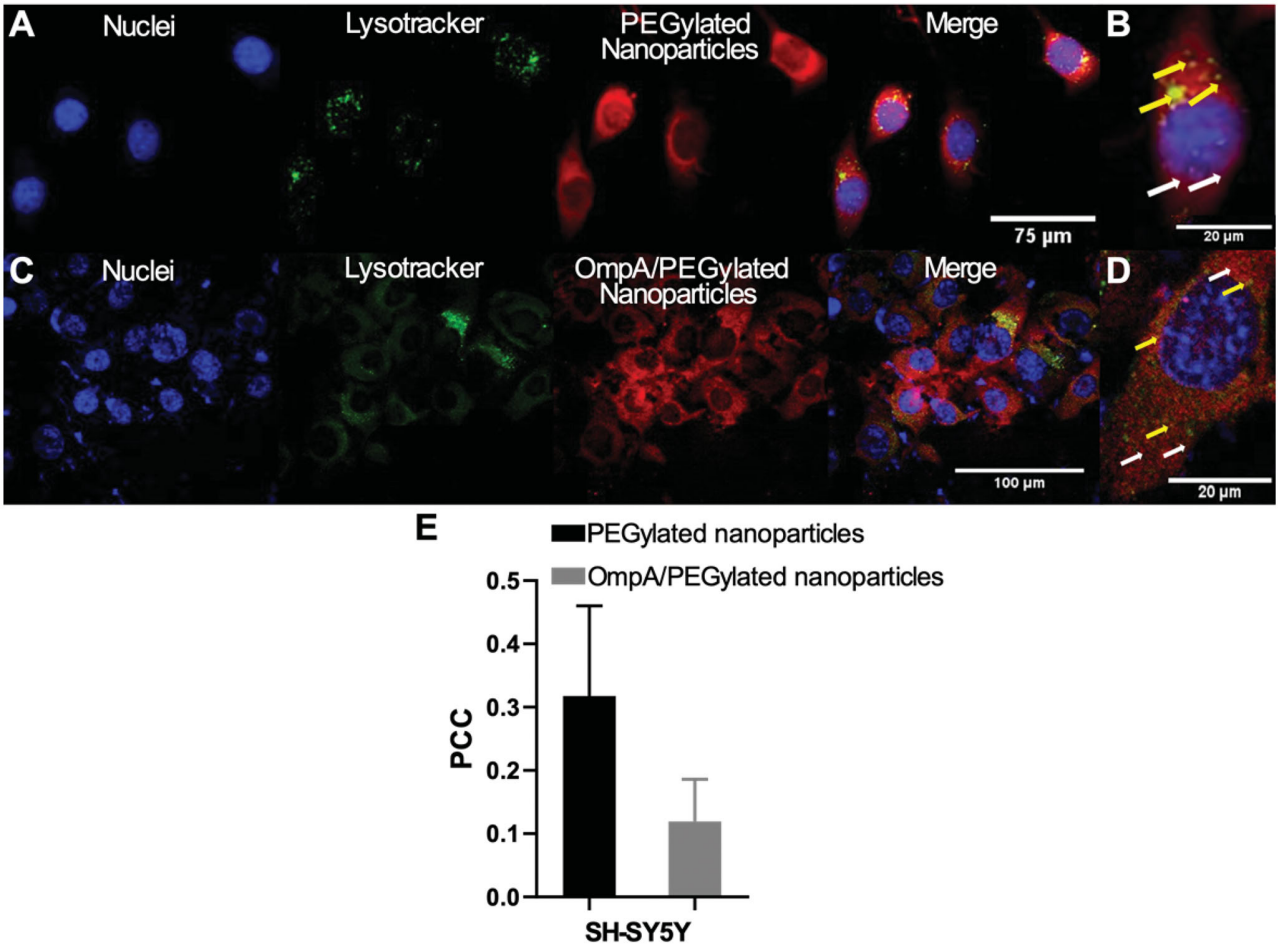
Endosomal escape of PEGylated nanoparticles and OmpA/PEGylated nanoparticles in SH-SY5Y cells. Images correspond to 2 h of exposure. (A) PEGylated nanoparticles and (C) OmpA/PEGylated nanoparticles. The first two channels correspond to nuclei (blue) and endosomes (green) labeled with Hoechst 33342 and Lysotracker green DND-26, respectively. The third channel corresponds to nanoparticles labeled with rhodamine-B (red) and the fourth to the merge of the first three channels. (B) and (D) correspond to enlarged view of SH-SY5Y cell from (A) and (C), respectively. The white arrows indicate endosomal escape areas (red areas), while the yellow ones showed colocalization of the nanoparticles and endosomes where no endosomal escape was achieved (yellow areas). (E) Pearson’s correlation coefficient (PCC), for both PEGylated nanoparticles and OmpA/PEGylated nanoparticles in SH-SY5Y cells after 2 h exposure. The significant decrease of PCC in the OmpA/PEGylated nanoparticles compared to the PEGylated nanoparticles indicates an important improvement in the endosomal escape capacities most likely due to the presence of the potent translocating molecule OmpA.

To assess the cellular uptake and the endosomal escape of PEGylated nanoparticles and OmpA/PEGylated nanoparticles, nanocarriers were labeled with rhodamine B and subsequently delivered to SH-SY5Y cells. Figure 5(A,C) shows confocal images of effective cellular internalization by PEGylated nanoparticles and OmpA/PEGylated nanoparticle as evidenced by the evenly distributed fluorescence signals (third channel). Colocalization of the labeled nanoparticles with Lysotracker Green DND-26 was studied through Pearson’s correlation coefficient (PCC) in order to determine the endosomal escape abilities of each vehicle. In the case of PEGylated nanoparticles, the PCC approached 0.318 ± 0.143 while that of the OmpA/PEGylated nanoparticles was 0.119 ± 0.067 (Figure 5(E)). These results indicate an approximate endosomal escape efficiency of 68% for the PEGylated nanoparticles and 88% for OmpA/PEGylated nanoparticles. Figure 5(B,D) shows an enlarged view of a single SH-SY5Y cell extracted from Figure 5(A,C), respectively. In these images, it is possible to observe endosomal escape areas (no colocalized red areas indicated with white arrows), and colocalization of the nanoparticles with Lysotracker that confirms no endosomal escape (indicated with yellow arrows). In the case of PEGylated nanoparticles, endosomal escape is strongly attributed to the proton-sponge effect (van den Boorn et al., 2011), while in OmpA/PEGylated nanoparticles endosomal escape can be attributed to the proton-sponge effect but also, to the membrane translocation abilities of the OmpA (Lopez-Barbosa et al., 2020). These findings demonstrated the potency of OmpA as a high-efficiency endosomal escape agent that can lead to a significant increase in the transfected siRNA. This will ultimately lead to more effective gene therapies for the treatment of Alzheimer’s and other neurodegenerative conditions.

## Discussion

Gene delivery techniques provide a promising strategy for the development of new treatments for neurogenerative diseases such as Alzheimer’s disease. Our study aimed to show the suppression of the *BACE1* gene expression in HFF-1 cells upon delivery of a specific siRNA conjugated on magnetite nanoparticles as carriers. We were interested in assessing whether the immobilization scheme influenced the efficiency of siRNA delivery and if it represented a risk for cellular viability. The therapeutic potential of BACE1 silencing has been proposed several times due to its direct association with neurodegeneration and accumulation of APP products (Singer et al., 2005; Sun et al., 2006; Wang et al., 2018) during early stages of Alzheimer’s disease. Despite the attractive potential of this approach, the stability of the siRNA molecules as well as its controlled delivery remains challenging.

We immobilized a specific siRNA strand on magnetite nanoparticles with the purpose of silencing BACE1 expression. This was intended to have the ability to monitor, control, and target the carrier to specific organs, tissues, or cells. The conjugate was PEGylated with the intention of increasing the stability and biocompatibility of the system, as well as to make it more hydrophilic to prevent cellular clearance upon delivery. Immobilized siRNA nanoconjugates exhibited low acute and chronic cytotoxicity in HFF-1 cells as measured by MTT assay (Figures 3(A,B)). Additionally, they were able to diminish BACE1 expression after cellular incubation (Figure 3(C)). In parallel, OmpA protein was co-immobilized on PEGylated nanoparticles to study enhanced endosomal escape strategies that could lead to an improved gene delivery system. OmpA/PEGylated nanoparticles exhibited high biocompatibility in SH-SY5Y cells (Figures 4(C,D)), high hemocompatibility (Figure 4(A)), and low thrombogenic effect (Figure 4(B)).

Delivery of siRNA to brain cells remains a challenge due to the low transfection efficacy and difficulties crossing of the blood–brain barrier that is usually encountered due to its large molecular weight (∼13 kDa) and strongly negatively charged backbone (Kim & Kim, 2009; David et al., 2010). We expect that due to the small size of the conjugates, ranging between 4 and 6 nm in diameter (Figure 1(E)), they can trespass the blood–brain barrier as well as penetrate brain cell membranes (Jain, 2012). In addition, magnetite nanoparticles provide the capability of controlling and imaging the exact position of the conjugate by means of a weak magnetic field, which is crucial in target delivery applications (Stephen et al., 2011).

Cellular internalization and endosomal escape abilities of PEGylated nanoparticles and OmpA/PEGylated nanoparticles were studied by confocal imaging. PEGylated nanoparticles exhibit high cellular uptake, usually reported as an internalization via an endocytic pathway, and a significant endosomal escape which is mainly due to the proton-sponge effect (van den Boorn et al., 2011). In brief, endocytosed conjugates are protonated at the polymer backbone due to the acidic environment within the endosome. These protonated tertiary amines generate a net influx of chloride ions, which continue to be absorbed by the polymer and lead to rapid swelling and rupture of the endosomes (Li et al., 2012). Additionally, OmpA/PEGylated nanoparticles exhibit enhanced endosomal escape abilities compared to the PEGylated nanoparticles. This can be related to an additional endosomal escape strategy known as the translocation mechanism, in which a molecule, typically a cationic peptide or a protein, induces pore formation when it self-assembles across the membrane of the endocytic vesicles generating membrane nanoruptures (Selby et al., 2017). Alternatively, such molecules might be able to induce local endosomal stress that ultimately leads to membrane destabilization (Nair et al., 2012).

After exposure to the reducing environment of the cellular cytoplasm, the disulfide bond between the conjugate and the siRNA molecule is broken (Yang et al., 2014). At this point, siRNA molecules are free to reach the Risk complex in the cytoplasm and therefore promote silencing of the *BACE1* gene (Alvarez-Erviti et al., 2011). In addition, due to the relatively short half-life of BACE1 protein, a change at the genomic level will rapidly induce a change in the amount of protein expression (Zhang et al., 2012). The results obtained in this study provide an important basis for the development of future vehicles for the delivery of siRNA improving and scaling its effectiveness in preclinical mice models of neurological diseases, with the potential to be exploited in Alzheimer’s disease therapeutics and even be extended to other degenerative diseases.

Immobilization of siRNA on PEGylated magnetite nanoparticles appears to maintain the biological activity of the molecules as evidenced by the successful silencing of *BACE1* gene in HFF-1 cells. This approach showed no significant impact on cytotoxicity while providing a suitable avenue for improving stability and wettability due to the presence of the highly hydrophilic PEG molecules on the surface of the conjugates. Due to their small size, if eventually delivered intravenously, siRNA conjugates will be most likely capable of trespassing the blood–brain barrier as well as being endocytosed by brain cells. Endosome compartments will be then escaped most likely by means of the proton-sponge effect or by a translocation mechanism if the vehicle is enhanced by the co-immobilization of the translocating protein OmpA. The results obtained in this study provide an important basis for the development of vehicles for the delivery of siRNA that can be exploited in Alzheimer’s disease therapeutics. Further experiments will be then focused on delivering the nanoconjugates into the primary neuron and astrocyte co-cultures to be able to confirm and understand the mechanisms of internalization, trafficking, and fate. Moreover, such experiments will be helpful to estimate more precisely the rates of endosomal escape as well as the different parameters involved in achieving superior silencing.
